# Anisotropic Platinum Nanoparticle-Induced Cytotoxicity, Apoptosis, Inflammatory Response, and Transcriptomic and Molecular Pathways in Human Acute Monocytic Leukemia Cells

**DOI:** 10.3390/ijms21020440

**Published:** 2020-01-09

**Authors:** Sangiliyandi Gurunathan, Muniyandi Jeyaraj, Hyeonwoo La, Hyunjin Yoo, Youngsok Choi, Jeong Tae Do, Chankyu Park, Jin-Hoi Kim, Kwonho Hong

**Affiliations:** Department of Stem Cell and Regenerative Biotechnology and Humanized Pig Center (SRC), Konkuk Institute of Technology, Konkuk University, Seoul 05029, Korea; gsangiliyandi@yahoo.com (S.G.); muniyandij@yahoo.com (M.J.); hyunwoo1001@naver.com (H.L.); hyunjinyoo7@gmail.com (H.Y.); choiys3969@konkuk.ac.kr (Y.C.); dojt@konkuk.ac.kr (J.T.D.); chankyu@konkuk.ac.kr (C.P.); jhkim541@konkuk.ac.kr (J.-H.K.)

**Keywords:** platinum nanoparticles, oxidative stress, mitochondrial dysfunction, endoplasmic reticulum stress, apoptosis, proinflammatory response, transcriptomic analysis, molecular pathway analysis

## Abstract

The thermoplasmonic properties of platinum nanoparticles (PtNPs) render them desirable for use in diagnosis, detection, therapy, and surgery. However, their toxicological effects and impact at the molecular level remain obscure. Nanotoxicology is mainly focused on the interactions of nanostructures with biological systems, particularly with an emphasis on elucidating the relationship between the physical and chemical properties such as size and shape. Therefore, we hypothesized whether these unique anisotropic nanoparticles could induce cytotoxicity similar to that of spherical nanoparticles and the mechanism involved. Thus, we synthesized unique and distinct anisotropic PtNPs using lycopene as a biological template and investigated their biological activities in model human acute monocytic leukemia (THP-1) macrophages. Exposure to PtNPs for 24 h dose-dependently decreased cell viability and proliferation. Levels of the cytotoxic markers lactate dehydrogenase and intracellular protease significantly and dose-dependently increased with PtNP concentration. Furthermore, cells incubated with PtNPs dose-dependently produced oxidative stress markers including reactive oxygen species (ROS), malondialdehyde, nitric oxide, and carbonylated protein. An imbalance in pro-oxidants and antioxidants was confirmed by significant decreases in reduced glutathione, thioredoxin, superoxide dismutase, and catalase levels against oxidative stress. The cell death mechanism was confirmed by mitochondrial dysfunction and decreased ATP levels, mitochondrial copy numbers, and PGC-1α expression. To further substantiate the mechanism of cell death mediated by endoplasmic reticulum stress (ERS), we determined the expression of the inositol-requiring enzyme (IRE1), (PKR-like ER kinase) PERK, activating transcription factor 6 (ATF6), and activating transcription factor 4 ATF4, the apoptotic markers p53, Bax, and caspase 3, and the anti-apoptotic marker Bcl-2. PtNPs could activate ERS and apoptosis mediated by mitochondria. A proinflammatory response to PtNPs was confirmed by significant upregulation of interleukin-1-beta (IL-1β), interferon γ (IFNγ), tumor necrosis factor alpha (TNFα), and interleukin (IL-6). Transcriptomic and molecular pathway analyses of THP-1 cells incubated with the half maximal inhibitory concentration (IC_50)_ of PtNPs revealed the altered expression of genes involved in protein misfolding, mitochondrial function, protein synthesis, inflammatory responses, and transcription regulation. We applied transcriptomic analyses to investigate anisotropic PtNP-induced toxicity for further mechanistic studies. Isotropic nanoparticles are specifically used to inhibit non-specific cellular uptake, leading to enhanced in vivo bio-distribution and increased targeting capabilities due to the higher radius of curvature. These characteristics of anisotropic nanoparticles could enable the technology as an attractive platform for nanomedicine in biomedical applications.

## 1. Introduction

Over the past decade, both academia and industry became interested in the development of novel nanoparticles (NPs) to detect and/or treat cancer and other infectious diseases [1,2], and efforts were directed toward the development of unique nano-sized materials. Due to their enhanced surface area-to-volume ratio, NPs have unique physicochemical properties and characteristic features compared with their bulk counterparts, such as augmented catalytic potential, distinctive plasmonic signatures, and enhanced transport capabilities that could be suitable for medical and industrial applications. Inorganic, notably metal NPs attracted research interest due their convenience and potential for specific targeting and sustained release [3]. Biologically synthesized NPs can be used for various purposes, but they are associated with undesirable side effects and solubility. Among various types of NPs, platinum NPs (PtNPs) have thermoplasmonic and catalytic potential, and they can serve as a source for the development of nano-based materials [4]. Platinum NPs are included in cosmetics, supplements, food additives, electrocatalytic processes, data storage systems, electronic devices, electrochemical biosensors, and chemical, fluorescence, and refractometry sensors [5,6,7]. Furthermore, PtNPs have biomedical applications as diagnostic mediators, medical implants, drug delivery vehicles, and photothermal therapy as they are less cytotoxic than other metal nanoparticles such as silver [8,9,10,11]. The biological responses of PtNPs depend on their physicochemical properties, including size, shape, surface moiety, core composition, and morphology [12]. Several studies showed that responses to NPs in vitro include cellular death, the activation of stress, inflammatory, and immune responses, cytotoxicity, genotoxicity, developmental abnormalities, the modification of gene transcription, and the modulation of signal transduction pathways [13,14]. Notably, PtNPs are both biocompatible with and cytotoxic to various types of human cells [15,16,17,18]. For instance, PtNPs can act as scavengers of reactive oxygen species (ROS) [19,20], and they induce developmental alterations and increase heart rates in zebrafish [21]. Ultra-small PtNPs induce cellular stress, cytotoxicity, DNA damage, and genotoxicity in vivo and in vitro in human monocytic [22], U2OS [18], and prostate cancer (LNCaP) cell lines [17].

Platinum-based NPs with attractive electrocatalytic properties [23,24] can function as catalysts and have therapeutic applications. They induce apoptosis by targeting specific signaling pathways [25], and they induce cytotoxicity in human colon carcinoma HT29 and Caco-2 cell lines, as well as the death of cervical cancer cells through mitotic G2 phase (G2/M) phase arrest [5,26]. Furthermore, PtNPs induce DNA damage in human cancerous and normal cells including colon cancer [27], monocytic [22], U2OS [18], cancer (LNCaP) [17], trophoblasts [28], and bronchial epithelial [29] cells. They also induce apoptosis through G0/G1 cell cycle arrest in the human breast adenocarcinoma cell line, MCF-7 [30]. Platinum NPs inhibit the activity of Taq DNA polymerase and damage DNA structures [31]. Human bronchial epithelium (BEAS-2B) and human lung alveolar type II epithelioid cells (A549) lose viability when incubated with PtNPs, and interleukin-8 (IL-8) concentrations increase remarkably [32]. Platinum NPs induce size-dependent toxicity in neuroblastoma, MDA-MB-231, and LNCaP cells, indicating that the diameter of these NPs plays a crucial role in cytotoxicity [33]. Graphene oxide and vanillin-functionalized graphene oxide induce pro-inflammatory cytokines in a human acute monocytic leukemia cell line [22], and PtNPs dose-dependently induce cytokines such as IL-1β, IL-8, and tumor necrosis factor alpha (TNFα) [34]. 

Findings of PtNP-induced reactions in cellular systems suggest that further evaluation is required to gain a deeper understanding of the biological responses induced by PtNPs, particularly those of an anisotropic nature, to ensure safety when applied in vivo. Biological PtNPs are needed to explore transcriptomic and molecular pathways in THP-1 cell lines. Transcriptome sequencing (RNA-seq) is a powerful tool for investigating genes and proteins at the levels of transcription and translation, respectively, as well as the molecular mechanisms of THP-1 after incubation with PtNPs. It is particularly useful for studying routinely applied nanoparticles such as PtNPs, since RNA-seq allows global examination of biological responses through gene expression. The expression of genes involved in metal metabolism was disrupted, as well as that of genes acting in transcription regulation and DNA binding, and clusters of genes associated with protein synthesis and structure are altered in THP-1 cells incubated with zinc oxide nanoparticles [35]. Several research groups investigated the toxicity of PtNPs because of their broad range of applications, but most of them were qualitative, showing, for example, that PtNPs induce cell death. However, a comprehensive approach to PtNP-induced cellular toxicity, apoptosis, and immunomodulation, and the transcriptome and molecular pathways involved in these effects of anisotropic PtNPs in THP-1 was not undertaken. Macrophages generally play critical roles in innate immune defense, having a powerful phagocytic capacity, and residing in many tissues. Such features render them appropriate for investigations into the toxicological impact of NPs in vitro [35,36]. Generally, nanoparticles are highly toxic. The toxic effect may be caused by their unique physical and chemical properties, which underlie specific mechanisms of interaction with living systems. In general, this determines the importance of studying the causes and mechanisms of the potential toxic effect of NPs. Normally, anisotropic nanoparticles have enhanced physical, chemical, and biological properties compared to spherical nanoparticles, including increased targeting avidity and decreased non-specific in vivo clearance compared to spherical nanoparticles. 

With these desirable and specific features of PtNP properties, anisotropic nanoparticles were successfully utilized to study biological effects in many biomedical settings, and these particles seem to be better than analogous spherical nanoparticles. Therefore, we evaluated anisotropic PtNP-induced cytotoxicity, apoptosis, and pro-inflammatory responses, and analyzed transcriptomic and molecular pathways in the human acute monocytic leukemia cell line THP-1. 

## 2. Results and Discussion

### 2.1. Synthesis and Characterization of PtNPs Using Lycopene

The main aim of PtNP synthesis using lycopene was to create non-toxic, secondary plant metabolites that are environmentally friendly and non-pollutant. Various types of NPs were synthesized using extracts of plant and microbes, as well as pure proteins, amines, amino acids, phenols, sugars, ketones, aldehydes, and carboxylic acids. We synthesized NPs in aqueous mixtures of 1 mM H_2_PtCl_6_∙6H_2_O and 1 mg/mL lycopene at 70 °C for 1 h in sealed flasks to avoid evaporation. Identical amounts of platinum and lycopene solutions were separately maintained under the same reaction conditions as controls. 

**Figure 1:**
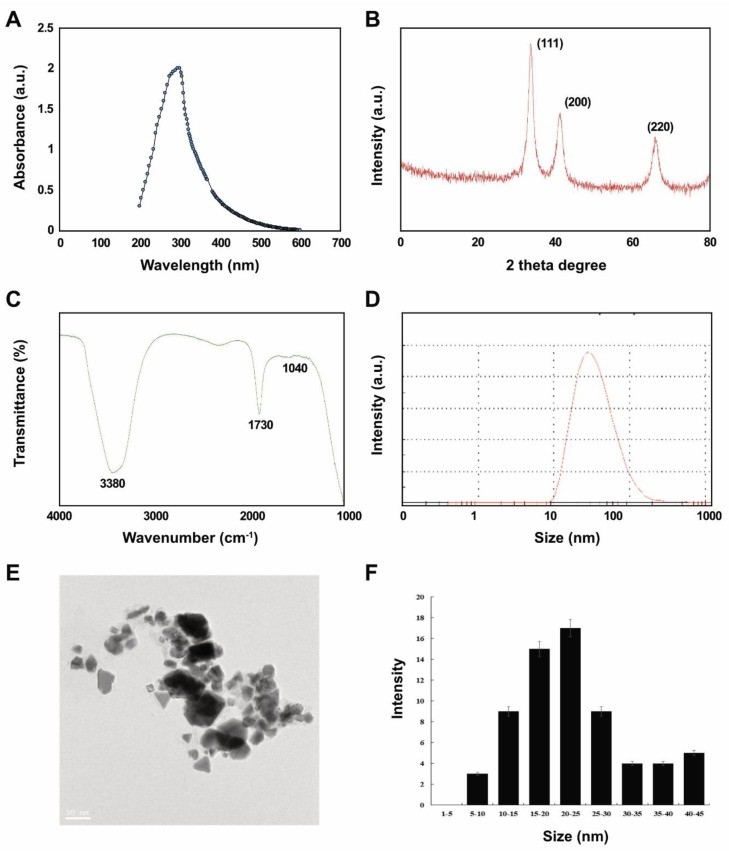
Synthesis and characterization of platinum nanoparticles (PtNPs) using lycopene. (**A**) Absorption spectra of lycopene-mediated synthesis of PtNPs. (**B**) X-ray diffraction (XRD) patterns of PtNPs. (**C**) Fourier-transform infrared (FTIR) spectra of PtNPs. (**D**) Size distribution analysis of PtNPs using dynamic light scattering (DLS). (**E**) TEM images of PtNPs. (**F**) Histograms showing particle size distribution. At least three independent experiments were performed for each sample, and reproducible results were obtained. The data represent the results of a representative experiment.

We initially confirmed that reduced Pt ions were incorporated into PtNPs as a change of color from yellow to dark brown, indicating the formation of palladium NP, and we then characterized the PtNPs using ultra-violet (UV)-visible light spectroscopy. An absorption peak in the 275–325 nm range of the UV-visible spectrum was characteristic of the surface plasmon resonance (SPR) band of PtNPs (Figure 1A). This suggested that lycopene reduced Pt ions and formed stable PtNPs. Others also showed that polyvinylpyrrolidone (PVP) [37], apigenin [22], and tangeretin [18] can reduce Pt and stabilize PtNPs. 

Figure 1B shows that crystalline PtNPs had the features of a Pt face-centered cubic (fcc) phase. First (111), second (200), and third (220) peaks corresponded to 2θ values of 41.9°, 46.5°, and 67.5°, respectively. The X-ray diffraction (XRD) peaks of PtNPs were broad and comparable to those of the corresponding bulk Pt material. The average size of Pt nanocrystallites was calculated from each XRD peak using the width of reflection according to the Debye-Scherrer equation. The particle size of polyhedral and anisotropic PtNPs was analyzed using transmission electron microscopy (TEM) to ensure a nanosized particle range of 5–45 nm. Over 90% of the PtNPs were polyhedral (spheres, cubes, rectangles, triangles, octahedrons, and tetrahedral or truncated cubic hexagons, octahedrons and tetrahedrons) with very sharp corners, edges, and facets. 

We then aimed to identify biomolecules in lycopene that might be responsible for reducing chloroplatinic acid ions and capping NP surfaces using Fourier-transform infrared spectroscopy (FTIR). The capping reagent responsible for the stability of the biomolecule reduced PtNPs. The interaction between PtNPs and lycopene generated peaks at 1730 and 3380 cm^−1^. Relative shifts in position and intensity distribution were confirmed by FTIR. This showed that the proteins capped the surface of the nanoparticles and stabilized them for longer periods. Figure 1C shows that the IR bands at 1730 cm^−1^ and 3380 cm^−1^ were characteristic of the C=O stretching mode of the carboxylic acid group and the O–H stretching mode, respectively [38,39]. The band due to C–O stretching merged into a very broad envelope centered on 1040 cm^−1^, arising from C–O–C symmetric stretching and the C–O–H bending vibrations of lycopene. The C=O stretching mode indicated that a –COOH group in the material bound to PtNPs. Thus, the bands at 1730 and 1040 cm^−1^ in FTIR indicated that PtNPs bind to proteins via free amine groups.

The average size of synthesized PtNPs measured as dynamic light scattering was 30 (range, 10–100) nm, which was slightly larger than that determined by TEM due to Brownian motion of the suspended particles (Figure 1D). The mean hydrodynamic diameter of PtNPs was 25.5 ± 5 nm with a polydispersity index of 0.150 ± 0.015. Furthermore, we performed size distribution analysis in various solutions such as water, Roswell Park Memorial Institute Medium (RPMI) medium, and RPMI medium with 10% fetal bovine serum (FBS) using dynamic light scattering (DLS) assay. It was found that the average size of PtNPs was 25.5 ± 5, 60 ± 11.0, and 40 ± 5.0 nm in water, RPMI medium, and RPMI medium with 10% serum, respectively. The results suggest that the PtNPs particles dissolved in RPMI medium and RPMI medium with 10% FBS were slightly larger than particles dissolved in water. DLS results for particle size in solution indicated that the PtNPs tended to form agglomerates in RPMI medium without serum compared to with serum.

Visualization by TEM showed spherical, cubic, rectangular, triangular, octahedral, and tetrahedral or truncated cubic, hexagonal, octahedral and tetrahedral particles with an average size of 25 (range, 5–50) nm (Figure 1E,F), which would favor high catalytic performance and efficiency.

### 2.2. Effects of PtNPs on Cell Viability and Proliferation of THP-1 Cells

**Figure 2:**
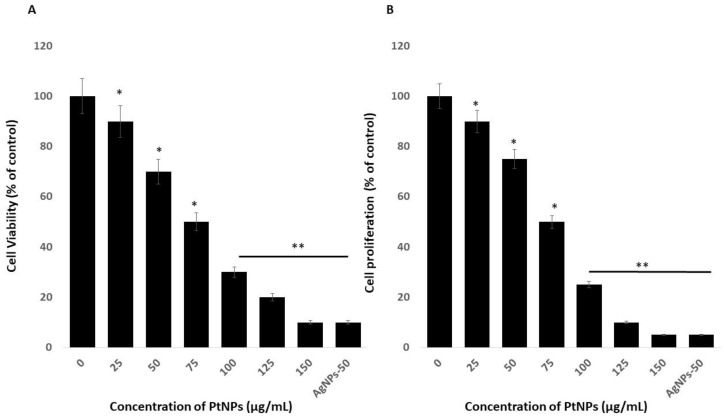
Effect of PtNPs on cell viability and proliferation of THP-1 cells. (**A**) Viability of THP-1 cells was determined after 24 h of exposure to different concentrations of PtNPs (25–150 µg/mL). (**B**) Proliferation of THP-1 cells was determined using the 5-bromo-2’-deoxyuridine (BrdU) assay after 24 h of exposure to different concentrations of PtNPs (25–150 µg/mL). The treated groups showed statistically significant differences from the control group by the Student’s *t*-test (* *p* < 0.05). * significant; ** highly significant.

The viability of THP-1 cells incubated with variously shaped PtNPs (25–150 μg/mL) for 24 h decreased dose-dependently with increasing concentrations of PtNPs. We then calculated the half-maximal inhibitory concentration (IC_50_) of PtNPs for THP-1 cells before analyzing transcriptomic and molecular pathways. Increasing PtNP concentrations corresponded to decreasing cellular metabolic activity (Figure 2A). The cells lost 10% and 90% of their viability when incubated with the minimal (25 μg/mL) and maximal (150 μg/mL) concentrations of PtNPs, compared with untreated cells that were 100% viable. The viability of THP-1 cells incubated with 25, 50, 75, 100, 125, and 150 μg/mL PtNPs was significantly decreased to 10%, 30%, 50%, 70%, 80%, and 90% respectively. The trend of THP-1 cell proliferation similarly decreased with increasing concentrations of PtNPs (Figure 2B). The IC_50_ of PtNPs against THP-1 cells was 75 μg/mL. Bendale et al. [15] reported that PtNPs exert cytotoxic effects on cancer cell lines, but not on normal cells, even at the highest dose tested. Increasing the ICR-191 concentration corresponded to decreasing cellular metabolic activity in non-cancerous HaCaT and cancerous MelJuSo cells incubated with PtNPs. In addition, ICR-191 has a more powerful impact on HaCaT than on MelJuSo cells [40]. PtNPs were established as having anticancer and cytotoxic activities against various type of cancerous and non-cancerous cells ha. For instance, PtNPs penetrate cells via diffusion or endocytosis and then form intracellular aggregates [5,41,42,43]. Platinum NPs inhibit HeLa cell viability and proliferation by activating p53 [41] and inhibiting U87, U118, and U251 glioblastoma tumor cell proliferation [41,44,45]. Cytotoxicity is dependent on PtNP size in Raw 264.7 cells incubated with various concentrations of PtNPs [46]. The present findings together with published data indicate that PtNPs would be excellent tools with which to analyze transcriptomic and molecular pathways.

### 2.3. Morphology of THP-1 Cells in the Presence of PtNPs

**Figure 3:**
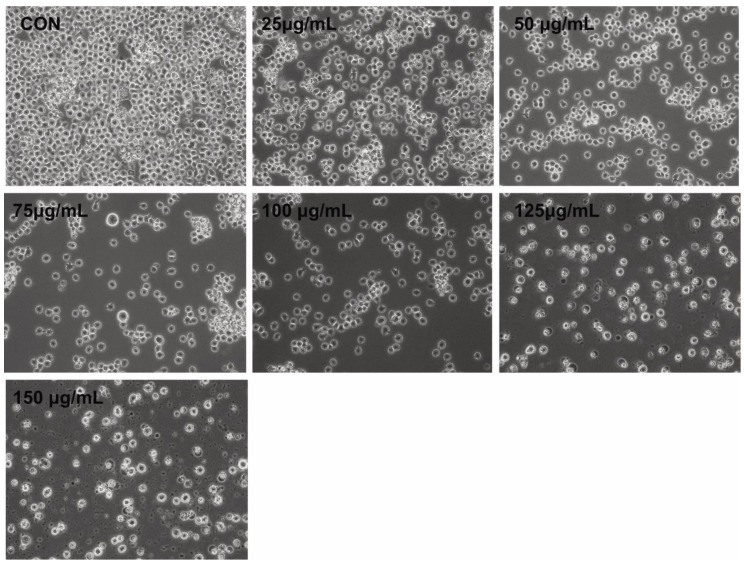
PtNPs altered the morphology of THP-1 cells. The effect of PtNPs on cell morphology was determined after 24 h of exposure to different concentrations of PtNPs (25–150 µg/mL) using an optical microscope. At least three independent experiments were performed for each sample. Scale bar, 200 µm.

Morphological changes comprise the hallmark of apoptosis. We assessed the effects of PtNPs on THP-1 cells to determine correlations between altered cell morphology and cell viability, as well as proliferation. The cells were incubated with PtNPs (25–150 μg/mL) for 24 h at the monocytic stage. Phase contrast microscopy revealed significant differences between control THP-1 macrophages and THP-1 cells incubated with PtNPs (Figure 3). The morphology of the cells incubated with various concentrations of PtNPs significantly changed, with loss of uniformity and remarkable shrinkage around cell clusters. Increasing PtNP concentrations caused remarkable changes in cell morphology such as extreme shrinkage, membrane blebbing, and loss of plasma membrane integrity. These results were consistent with those of cell viability, cell proliferation, and lactate dehydrogenase (LDH) and intracellular protease leakage. Digital microscopy data suggested that PtNPs are cytotoxic. Kutwin et al. [44] reported that the morphology of U87 glioblastoma cells incubated with various concentrations of PtNPs was characteristic of cell death with long branched protrusions and shrinkage. Collectively, PtNPs induced morphological changes that eventually led to cell death.

### 2.4. PtNPs Induce Cytotoxicity in THP-1 Cells

**Figure 4:**
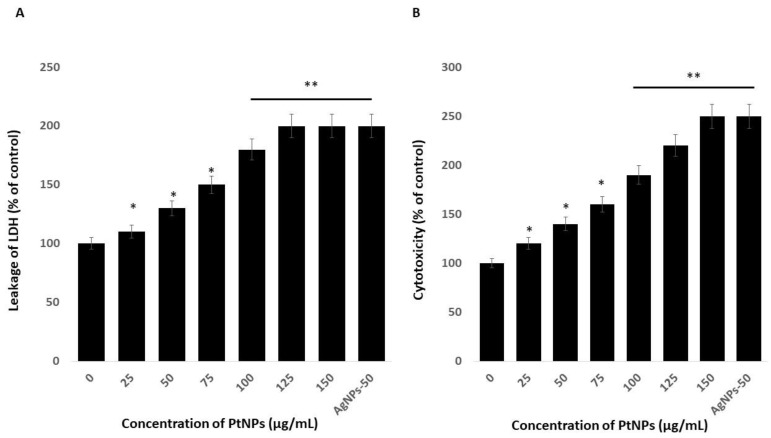
PtNPs increase the leakage of lactate dehydrogenase (LDH) and intracellular protease. (**A**) THP-1 cells were treated with PtNPs (25–150 µg/mL) for 24 h, and LDH activity was measured at 490 nm using the LDH cytotoxicity kit. (**B**) Intracellular protease activity was determined by assessing the association of intracellular proteases with a luminogenic peptide substrate (alanyl-alanylphenylalanyl-aminoluciferin) after 24 h of exposure to PtNPs (25–150 µg/mL). Cell death was quantified as the ratio of living cells. At least three independent experiments were performed for each sample. Results are expressed as mean fold change ± standard deviation from three independent experiments. The treated groups showed statistically significant differences from the control group by the Student’s *t*-test (* *p* < 0.05; ** *p* < 0.01). * significant; ** highly significant.

Platinum nanoparticles were significantly cytotoxic to THP-1 cells. Cytotoxicity induces cell death upon membrane disruption that leads to release of the cytosolic enzyme lactate dehydrogenase (LDH) into the extracellular medium. We found more LDH in the medium of cells incubated with PtNPs than in controls, indicating that NPs lyse cells [22]. Figure 4A shows more LDH leakage at 75–150 µg/mL PtNPs, indicating a significant decrease in cell viability and a significant increase in LDH release. These findings indicated that the decreased cell viability and damage caused by the NPs ultimately caused the death of the cells [47]. Others also reported that PtNPs induce cytotoxicity through LDH leakage in cancer cells including A549 lung carcinoma, LNCaP prostate cancer, and OS epithelial (U2OS) cells [17,18,47]. 

We assessed associations between intracellular proteases and a luminogenic peptide substrate in membrane integrity assays to further confirm the cytotoxic nature of medium-sized, anisotropic PtNPs in THP-1 cells. The results were in agreement with those of the LDH assays; increasing concentrations of PtNPs increased intracellular protease leakage via membrane damage (Figure 4B). The LDH leakage and intracellular protease caused more membrane damage and, consequently, more LDH enzyme and protease in the medium with respect to corresponding PtNP concentrations.

### 2.5. Platinum-Based NPs Induce ROS, Lipid peroxidation (LPO), Nitric Oxide (NO), and Protein Carbonylation in THP-1 Cells

**Figure 5:**
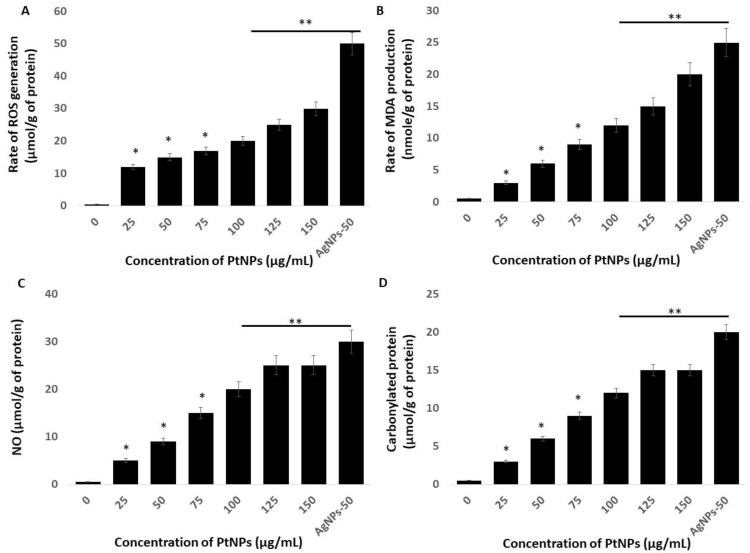
PtNPs increase generation of reactive oxygen species (ROS), lipid peroxidation, nitric oxide (NO), and protein carbonylation (**A**) THP-1 cells were treated with PtNPs (25–150 µg/mL) for 24 h. Spectrophotometric analysis of ROS using DCFH-DA. (**B**) Malondialdehyde (MDA) concentration was measured using a thiobarbituric acid-reactive substances assay and is expressed as nanomoles per gram of protein. (**C**) NO production was quantified spectrophotometrically using the Griess reagent and is expressed as micromoles per gram of protein (**D**). Protein carbonylation content was determined and is expressed as micromoles per gram of protein. The results are expressed as means ± standard deviation of three independent experiments. The treated groups showed statistically significant differences from the control group by the Student’s *t*-test (* *p* < 0.05; ** *p* < 0.01). * significant; ** highly significant.

The link between oxidative stress and the redox balance plays critical roles in the cellular and tissue functions of cells and tissues and their impairment leads to various diseases. Macromolecular oxidation in response to ROS is associated with impaired cellular functions. Reactive nitrogen species (RNS) elicit various modifications of macromolecules and lead to nitro-oxidative stress. We investigated oxidative, lipoperoxidation, nitro-oxidative stress, and protein carbonylation in THP-1 cells incubated with various concentrations of PtNPs for 24 h. Figure 5A shows that PtNPs dose-dependently induced intracellular ROS production, with the response being substantial at 75 μg/mL. Platinum NPs induce ROS generation by impairing receptor activator of nuclear factor κB ligand (RANKL) signaling [48]. For instance, incubating HepG2 cells with 68-nm PtNPs suggested that ROS are dose-dependently induced [34]. Ultra-small PtNPs also induce ROS generation in THP-1 cells [22]. The increased amount of intracellular ROS due to PtNPs could affect the integrity of cell components. Altogether, our findings suggested that ROS production is a common mechanism of toxicity In Vitro and In Vivo. 

That PtNPs induced oxidative stress with lipid peroxidation was confirmed by the dose-dependent increase in malondialdehyde (MDA) in THP-1 cells incubated with PtNPs. The results showed a statistically significant increase in MDA with increasing doses of PtNPs (*p* < 0.05; Figure 5B). Exposure to PtNPs might cause the production of ROS that subsequently increase MDA concentrations. An imbalanced redox potential might also be a cause of PtNP toxicity. Administering PtNPs induces intracellular oxidative stress via the generation of ROS that disrupt the antioxidant system [18,47]. An imbalance in antioxidant enzymes also causes lipid peroxidation [49], which deteriorates the cell membrane and renders it susceptible to further oxidation by free radicals [49,50]. Excess ROS production results in the oxidation of cell components such as proteins and lipids. Increased amounts of hydroxyl peroxyl radicals disrupt double bonds in fatty acids and generate highly reactive radicals, which in turn increase MDA production. We concluded that an increased amount of MDA might disintegrate the cellular assembly by altering proteins, carbohydrates, and nucleic acid bases.

We investigated the role of NO in the PtNP-induced cytotoxicity of THP-1 cells. We found that incubating THP-1 cells with 25–150 μg/mL PtNPs for 24 h caused a concentration-dependent increase in NO (Figure 5C). The amounts of NO were 5 and ~25 μM in cells incubated with 25 and 150 μg/mL PtNPs compared with 0.5 μM of NO in untreated cells. The amount of NO was significantly augmented in THP-1 cells incubated with 25, 50, 75, 100, 125, and 150 μg/mL PtNPs to 5, 9, 15, 20, and 25 μM respectively. Silver NPs induce NO in various types of cancer cells including human pancreatic ductal adenocarcinoma and human alveolar basal epithelial cells [39,51]. Platinum NPs similarly induce NO in cancerous cells such as THP-1 and human prostate cancer cells [17,22]. The abnormal generation of NO increases nitro-oxidative stress and upregulation of the cell-death mediator p53 [52]. Collectively, PtNPs dose-dependently induce NO that is responsible for cell death induced by nitro-oxidative stress. 

Oxidative stress is the result of an imbalance between ROS generation and detoxification, and protein carbonyl levels might become enhanced as a consequence. Thus, how biological systems respond to oxidative stress induced by NP can be determined by measuring protein carbonylation, which is a biomarker of oxidation. Hence, we estimated protein carbonylation in THP-1 cells incubated with 25–150 μg/mL PtNPs for 24 h and found a dose-dependent increase in carbonylated proteins (Figure 5D). The amounts of protein carbonylation were 3 and ~15 μM in cells incubated with 25 and 150 μg/mL PtNPs, respectively, whereas that in untreated cells was 0.5 μM. Protein carbonylation was significantly increased to 3, 6, 9, 12, and 15 μM in THP-1 cells incubated with 25, 50, 75, 100, 125, and 150 μg/mL PtNPs, respectively. Silver NPs induce protein carbonylation in *Daphnia magna* [53], as well as in THP-1 macrophages, primary neuronal cells, and human colon epithelial cells in a particle size-dependent manner [54,55,56]. Carbonylation is irreversible; it causes a loss of protein function that is often associated with protein unfolding and aggregation, and it is involved in signal transduction [57]. Oxidatively modified proteins are involved in glucose metabolism, mitochondrial function, cellular motility/structural integrity, and protein degradation [58]. Elevated protein carbonylation causes various type of pathologies including metabolic [59], neurodegenerative [60] aging, and age-related diseases [61,62]. Collectively, these findings suggest that oxidative stress is a common mechanism of PtNP-induced toxicity in THP-1 cells through increasing intracellular amounts of ROS, MDA, NO, and protein carbonylation. 

### 2.6. Platinum NPs Impair Antioxidants in THP-1 Cells

**Figure 6:**
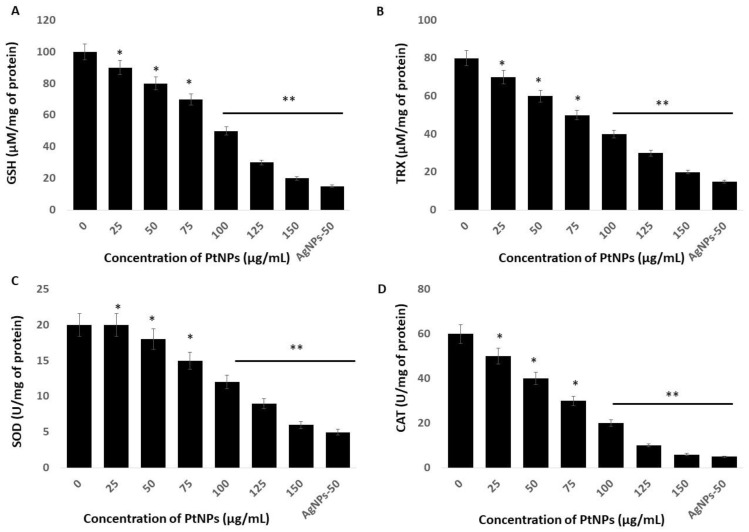
Effect of PtNPs on antioxidant markers. THP-1 cells were treated with different concentrations of PtNPs (25–150 µg/mL) for 24 h. After incubation, the cells were harvested and washed twice with ice-cold phosphate-buffered saline solution. The cells were collected and disrupted by ultrasonication for 5 min on ice. (**A**) Glutathione (GSH) concentration is expressed as µM per mg of protein. (**B**) Thioredoxin (TRX) concentration is expressed as µM per mg of protein. (**C**) Superoxide dismutase (SOD) is expressed as units per mg of protein. (**D**) Catalase (CAT) is expressed as units per mg of protein. Results are expressed as mean fold change ± standard deviation from three independent experiments. he treated groups showed statistically significant differences from the control group by the Student’s *t*-test (* *p* < 0.05; ** *p* < 0.01). * significant; ** highly significant.

Incubating cells with nanoparticles causes an imbalance of antioxidants in a cellular oxidative state by increasing ROS and decreasing antioxidants. Reduced glutathione (GSH) is an important intracellular antioxidant that plays vital roles in protecting cells against oxidative stress [63]. The amount of GSH decreases in cells under oxidative stress due to its oxidation to glutathione disulfide (GSSG). The amount of GSH was significantly reduced at all tested PtNPs concentrations (Figure 6A). Concentrations of intracellular GSH significantly decreased from 90 to ~20 μM in THP-1 cells incubated with 25 and 150 μg/mL PtNPs, respectively, compared with the 100 μM in untreated cells. The amount of GSH was significantly decreased to 90, 80, 70, 50, and 20 μM, respectively, in THP-1 cells incubated with 25, 50, 75, 100, 125, and 150 μg/mL PtNPs. These findings agreed with those of previous studies showing decreased GSH concentrations in HT29, A549 lung carcinoma, osteosarcoma, prostate cancer, and THP-1 cells incubated with PtNPs [17,22,27,47].

Thioredoxin (TRX) is a small 12-kDa protein with essential functions as an active oxidoreductase and an electron donor of peroxiredoxins that are important for peroxide reduction [64]. Hence, we evaluated the amounts of TRX in THP-1 cells incubated with PtNPs for 24 h and found a significant reduction in TRX at all tested concentrations of PtNPs (Figure 6B). Increasing concentrations of PtNPs induced a significant decrease in TRX. The amounts of TRX were 70 and ~20 μM in cells incubated with 25 and 150 μg/mL PtNPs, respectively, compared with 80 μM in untreated cells. The amounts of TRX were significantly decreased to 80, 70, 60, 50, 40, 30, and 20 μM, respectively, in THP-1 cells incubated with 25, 50, 75, 100, 125, and 150 μg/mL PtNPs. Thioredoxin regulates various cellular functions in response to redox signals and stress, and modulates various signaling pathways, transcription factors, and immunological responses [65]. It minimizes damage to vital organelles such as mitochondria and nuclei via ROS leakage during mitochondrial respiration [66]. Furthermore, cytosolic TRX plays important roles in the control of growth, apoptosis, and chronic inflammation [67]. Overall, PtNPs can play critical roles in decreasing intracellular concentrations of TRX, which is essential for many cellular functions.

We measured superoxide dismutase (SOD) protein in THP-1 cells incubated for 24 h with PtNPs and found a statistically significant decrease in SOD as the PtNP concentrations increased (Figure 6C). The level of SOD was 20 and ~6 μM in cells incubated with minimal (25 μg/mL) and maximal (150 μg/mL) concentrations of PtNPs compared with 20 μM in untreated cells. The amounts of SOD in THP-1 cells incubated with 25, 50, 75, 100, 125, and 150 μg/mL PtNPs were significantly decreased to 20, 18, 15, 12, 9, and 6 μM, respectively. Metal NPs such as AgNPs induce ROS and reactive nitrogen species (RNS) in PANC-1 cells in association with significantly disturbed antioxidant enzymes at the protein and messenger RNA (mRNA) levels. The production of ROS and/or RNS along with an impaired antioxidant system led to the programmed death of cancer cells [68]. Zinc oxide NPs (ZnONPs) significantly decrease antioxidant concentrations in hepatoma cells and trigger apoptosis [69]. A reduced amount of SOD leads to the apoptosis of human skin carcinoma and human fibrosarcoma cells incubated with 20 nm AgNPs [70,71], and amounts of SOD1 protein and mRNA are reduced in PANC-1 cells incubated with metal nanoparticles [51].

Others showed that PtNPs can interfere with cellular functions, cause toxic effects, and might interfere with specific biological systems [22,47]. Toxicity induced by PtNPs is the result of ROS generation, glutathione depletion, and alterations in the amounts of intracellular superoxide dismutase (SOD) and in catalase (CAT) enzyme activities. Catalase is a crucial antioxidant enzyme that plays a critical role in maintaining the intracellular redox balance. We measured CAT in THP-1 cells incubated with PtNPs. We found a statistically significant decrease in CAT after incubation with increasing PtNP concentrations (Figure 6D). The levels of CAT were 50 and ~6 μM in cells incubated with minimal (25 μg/mL) and maximal (150 μg/mL) concentrations of PtNPs, respectively, compared with 60 μM in untreated cells. The amount of CAT was significantly decreased to 50, 40, 30, 20, 10, and 6 μM, respectively, in THP-1 cells incubated with 25, 50, 75, 100, 125, and 150 μg/mL PtNPs. Intracellular oxygen reduced to water through electron transport chains protects cells from normal ROS damage caused by catalase. A decreased amount of catalase cannot overcome damage caused by oxidative stress. 

### 2.7. PtNPs Cause Mitochondrial Dysfunction, and Decrease ATP Content, Mitochondrial Copy Number, and Peroxisome Proliferator-Activated Receptor Gamma Coactivator 1-Alpha (PGC-1 Alpha) Expression

**Figure 7:**
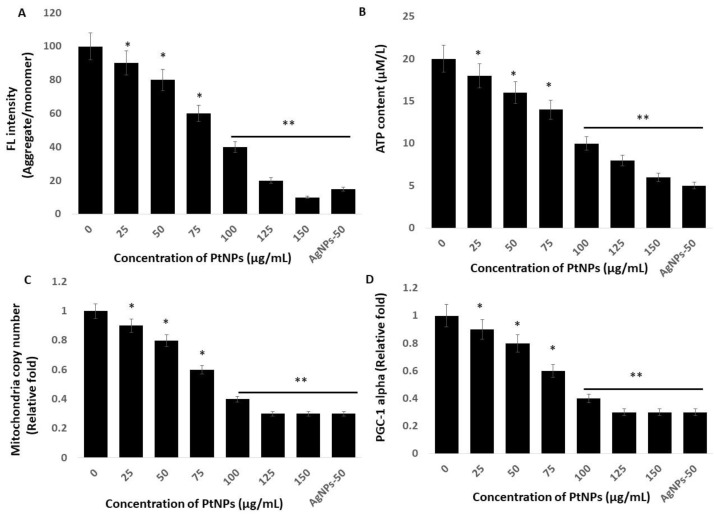
PtNPs decreased mitochondrial membrane potential (MMP), ATP content, mitochondrial copy number, and Peroxisome proliferator-activated receptor gamma coactivator 1-alpha (PGC-1 Alpha) PGC1α expression. (**A**) THP-1 cells were treated with PtNPs (25–150 µg/mL) for 24 h, and MMP was determined using the cationic fluorescent indicator cationic carbocyanine dye (JC-1). (**B**) THP-1 cells were treated with PtNPs (25–150 µg/mL) for 24 h, and the intracellular ATP content was determined according to the manufacturer’s instructions (Sigma-Aldrich, St. Louis, MO, USA; Catalog Number MAK135). (**C**). Mitochondrial copy number was determined via RT-PCR. (**D**). Expression of PGC1α was determined via RT-PCR. Results are expressed as mean fold change ± standard deviation from three independent experiments. The treated groups showed statistically significant differences from the control group by the Student’s *t*-test (* *p* < 0.05; ** *p* < 0.01). * significant; ** highly significant.

Mitochondria are the powerhouses of cells and crucial to the regulation of important functions such as ROS production and scavenging, ATP production, intracellular regulation of Ca^2+^ and apoptotic cell death, and activation of the caspase family of proteases [72]. Therefore, studies of the effects of PtNPs on mitochondrial dysfunction are essential. We investigated the integrity of the mitochondrial membrane by incubating THP-1 cells with various concentrations of PtNPs for 24 h and then measuring aggregate-to-monomer ratios. The intracellular amounts of monomers and the aggregate ratio significantly decreased with increasing concentrations of PtNPs (Figure 7A). The aggregate/monomer ratio was 90% and ~10% in cells incubated with minimal (25 μg/mL) and maximal (150 μg/mL) concentrations of PtNPs, respectively, compared with 100% in untreated cells. The aggregate-to-monomer ratios were significantly decreased to 90%, 80%, 60%, 40%, 20%, and 10%, respectively, in THP-1 cells incubated with 25, 50, 75, 100, 125, and 150 μg/mL PtNPs. Changes in mitochondrial membrane potentials indicate mitochondrial dysfunction, which ultimately decreases ATP production [73]. Others also suggested that PtNPs cause mitochondrial dysfunction in human embryonic kidney (HEK293) [74], THP-1 [22], prostate cancer [17], and osteosarcoma [18] cells through changing the membrane potential. Mitochondrial dysfunction induces apoptotic cell death through the loss of mitochondrial membrane potential (MMP), and increased expression of the Bcl-2 family of proteins promotes the release of cytochrome c, a major effector of apoptosis, which, together with the loss of MMP, causes early events in some apoptotic processes [75,76,77].

We assessed the amount of ATP produced in THP-1 cells incubated with various concentrations of PtNPs for 24 h, because ATP synthesis is dependent on the integrity of the mitochondrial membrane, which regulates hydrogen ion pumping across the inner membrane during electron transport and oxidative phosphorylation [78]. The results showed a statistically significant decrease in ATP production as the PtNPs concentration increased throughout the applied range (Figure 7B). The amount of ATP was 18 and ~6 μM in cells incubated with the minimal (25 μg/mL) and maximal (150 μg/mL) concentrations of PtNPs compared with 20 μM in untreated cells. Amounts of ATP were significantly decreased to 18, 16, 14, 10, 8, and 6 μM, respectively, in THP-1 cells incubated with 25, 50, 75, 100, 125, and 150 μg/mL PtNPs. Platinum NPs might decrease the production of ATP in human THP-1 [22], prostate cancer [17], and osteosarcoma [18] cells. Excessive ROS generation impairs mitochondrial functions in terms of diminished oxidative capacity and antioxidant defense, reduced *oxidative phosphorylation* (OXPHOS), and decreased ATP production [79], and it damages mitochondrial proteins/enzymes, membranes, and DNA, collectively leading to the interruption of ATP generation and other essential mitochondrial functions [80]. Overall, PtNPs play an important role in ATP production.

An imbalance between pro-oxidants and antioxidants causes oxidative damage to mitochondrial proteins, DNA, and lipids [81], which impairs enzyme functions in the respiratory chain and ultimately leads to mitochondrial dysfunction and reduced mitochondrial biogenesis [82]. Reduced numbers of mitochondria and a diminished capacity for oxidative phosphorylation results in impaired mitochondrial biogenesis [83,84]. Hence, we determined the copy number of mitochondria using RT-PCR and found that it significantly decreased in THP-1 cells incubated with increasing PtNP concentrations over the entire range of applied concentrations. (Figure 7C). The relative ratio of copy numbers was decreased 0.9- and ~0.3-fold in cells incubated with the minimal (25 μg/mL) and maximal (150 μg/mL) concentrations of PtNPs, respectively, compared with control. The fold copy number was significantly decreased 0.9-, 0.8-, 0.6-, 0.4-, 0.3-, and 0.3-fold, respectively, in THP-1 cells incubated with 25, 50, 75, 100, 125, and 150 μg/mL PtNPs. 

Mitochondrial biogenesis is controlled by several transcription factors that are important to maintain the number and size of mitochondria, as well as nuclear and mitochondrial genomes. Among several transcription factors, peroxisome proliferator-activated receptor (PPAR)-γ coactivator-1α (PGC-1α) plays a critical role in biogenesis. Therefore, we evaluated PGC-1α expression in THP-1 cells incubated with PtNPs for 24 h. The expression of PGC-1α was significantly and dose-dependently decreased by increasing concentrations of PtNPs (Figure 7D). The relative decrease was 0.9- and ~0.3-fold in cells incubated with the minimal (25 μg/mL) and maximal (150 μg/mL) concentration of PtNPs compared with control. The fold copy number was significantly decreased to 0.9-, 0.8-, 0.6-, 0.4-, 0.3-, and 0.3-fold, respectively, in THP-1 cells incubated with 25, 50, 75, 100, 125, and 150 μg/mL PtNPs, and this was associated with mitochondrial copy numbers. Therefore, THP-1 cells incubated with PtNPs were associated with defective mitochondrial biogenesis manifested by impaired mitochondrial dysfunction, reduced ATP generation, low copy numbers, and decreased mitochondrial gene expression.

### 2.8. PtNPs Induce Cell Death Mediated by Endoplasmic Reticulum Stress

**Figure 8:**
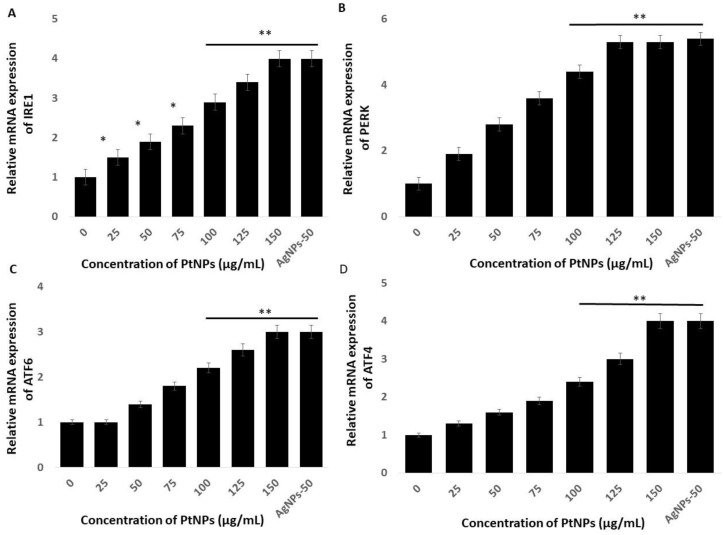
PtNPs enhance the expression of endoplasmic reticulum stress (ERS) genes. THP-1 cells were treated with various concentrations of PtNPs (25–150 µg/mL) for 24 h. The messenger RNA (mRNA) expression of ERS genes (**A**) inositol-requiring enzyme 1 (IRE1), (**B**) PKR-like ER kinase (PERK), (**C**) activating transcription factor 6 (ATF6), and (**D**) ATF4 was analyzed using quantitative reverse-transcription polymerase chain reaction. After 24 h, the fold change in the expression was determined relative to GAPDH expression. Results are expressed as mean fold change ± standard deviation from three independent experiments. The treated groups showed statistically significant differences from the control group by the Student’s *t*-test (* *p* < 0.05; ** *p* < 0.01). * significant; ** highly significant.

The endoplasmic reticulum (ER) regulates various cellular functions including the synthesis and post-translational modifications of secretory, luminal, and transmembrane proteins, protein folding, and the maturation of secretory and membrane proteins synthesized de novo [85]. Various external factors including NP, can trigger endoplasmic reticulum stress (ERS) which can result in aberrant protein folding. The unfolded protein response (UPR) is a is a component of the ER adaptive system that is highly conserved in most eukaryotes. The UPR comprises the principal signaling pathways that involve inositol-requiring enzyme 1 (IRE1), (PERK), and ATF6 [86]. All these genes play critical roles in the UPR adaptive system. We quantified the expression of the established ERS markers IRE1, PERK, ATF6, and ATF4, to determine the effects of PtNPs on ERS in THP-1 cells incubated with various concentrations of PtNPs for 24 h. Platinum NPs obviously induced all of these stress markers at all applied concentrations (Figure 8). These data showed that PtNPs cause ERS and associated UPR induction in THP-1 cells. Christen et al. [87] reported that silica NPs induce ERS through the activation of ATF-4, BiP, and X-box binding protein 1 (XBP-1s) in Huh7 cells. The *IRE1* gene induces apoptosis by activating apoptosis signaling kinase 1 (ASK1) and interacting with tumor necrosis factor receptor-associated factor (TRAF)2. Excessive and prolonged ER stress ultimately causes apoptosis by promoting the expression of CCAAT/enhancer-binding protein homologous protein (CHOP) [88,89,90]. Sustained and long-term ERS accelerates oxidative stress [90], which in turn accelerates ERS and activates apoptotic signaling pathways [91,92]. Overall, PtNPs induced ERS-mediated cell death through the expression of UPR genes responsible for adaptation in THP-1 cells.

### 2.9. PtNPs Induce Expression of Pro-Apoptotic and Anti-Apoptotic Genes 

**Figure 9:**
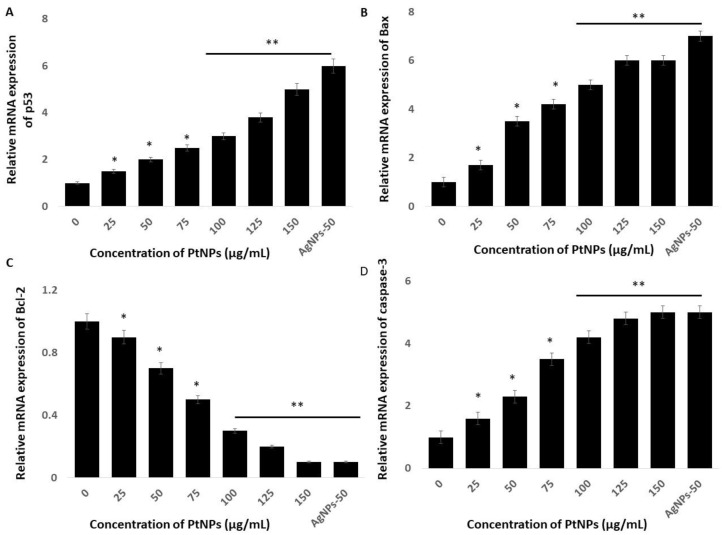
Effect of PtNPs on expression of pro- and anti-apoptotic genes. THP-1 cells were treated with various concentrations of PtNPs (25–150 µg/mL) for 24 h. The mRNA expression of (**A**) p53, (**B**) Bax, (**C**) Bcl-2, and (**D**) caspase-3 genes was analyzed using quantitative reverse-transcription polymerase chain reaction in THP-1 cells treated for 24 h with PtNPs. After 24 h, the fold change in the expression was determined relative to GAPDH expression. All experiments were performed in triplicate, each being repeated at least three times. The treated groups showed statistically significant differences from the control group by the Student’s *t*-test (* *p* < 0.05; ** *p* < 0.01). * significant; ** highly significant.

Cell fate depends on the balance between the extent/severity of ERS and the capacity of the ER to restore ER homeostasis through the UPR. Stress to the ER induces apoptosis via major extrinsic and intrinsic pathways [93]. Mitochondrial dysfunction might play critical roles in ERS and activate genes associated with apoptosis, such as the Bcl-2 family in particular. On the other hand, ER stress activates p53, which induces apoptosis [94,95,96]. Stress to the ER activates many genes involved in the control of cell fate, including anti-apoptotic and pro-apoptotic genes such as Bax and Bcl-2 [97,98]. Hence, understanding the roles and mechanisms of cell apoptosis induced by ERS in THP-1 cells incubated with PtNPs might provide important insights into how PtNPs induce cell death mediated by ER stress. Therefore, we evaluated expression of the important pro-apoptotic genes p53, Bax, Bcl-2, and caspase-3 in THP-1 cells incubated with various concentrations of PtNPs for 24 h. The results showed that PtNPs significantly activated these genes up to five-fold, and significantly downregulated Bcl-2 (Figure 9). The balance between anti- and pro-apoptotic proteins is important for maintaining normal mitochondrial function, as well as cell survival. The Bcl-2 family regulates ERS through physical interaction with ERS sensors and UPR components to induce apoptosis. Caspases also play crucial roles in ERS-mediated apoptosis. For instance, caspase-12 is activated during ERS, which sequentially activates caspase-7 and/or caspase-3, leading to mitochondria-independent apoptosis [99]. Our findings indicated that PtNPs respectively activate and downregulate pro- and anti-apoptotic genes, consequently leading to cell death. Collectively, ERS plays a critical role in PtNP-induced apoptosis that is mediated by mitochondria.

### 2.10. PtNPs Induce Oxidative Damage to DNA 

**Figure 10:**
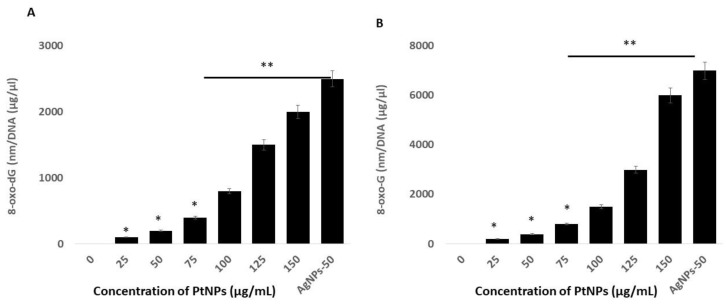
PtNPs increase oxidative damage to DNA and RNA. (**A**). THP-1 cells were treated with various concentrations of PtNPs (25–150 µg/mL) for 24 h. The 8-oxo-2′-deoxyguanosine (8-oxo-dG) level was measured after 24 h of exposure. (**B**). THP-1 cells were treated with various concentrations of PtNPs (25–150 µg/mL) for 24 h. The 8-oxoguanine (8-oxo-G) level was measured after 24 h of exposure. Results are expressed as means ± standard deviation from three independent experiments. There was a significant difference between treated cells and untreated cells as per Student’s *t*-test (* *p* < 0.05). The treated groups showed statistically significant differences from the control group by the Student’s *t*-test (* *p* < 0.05; ** *p* < 0.01). * significant; ** highly significant.

Reactive oxygen species are highly reactive, and they can oxidize lipids, proteins, and nucleic acids. The incorporation of an oxidized base can result in oxidative damage to mitochondrial DNA (mtDNA) and nuclear DNA (nDNA). The most prominent and susceptible modified nucleobase adducts are 8-oxo-2′-deoxyguanosine (8-oxo-dG) and 8-oxoguanine (8-oxoG), the latter of which is the major oxidized base in nucleotide pools, and in polymerized DNA or RNA [100,101]. Quantifying these adducts in THP-1 cells incubated with PtNPs might provide critical insights into the role of PtNPs in oxidative damage to mtDNA and normal DNA. Hence, we measured the levels of 8-oxo-dG and 8-oxoG using ELISA in THP-1 cells incubated with PtNPs for 24 h. Increasing concentrations of PtNPs induced more 8-oxo-dG and 8-oxoG accumulation, with the effect being more pronounced at higher concentrations (Figure 10). The concentrations of 8-oxo-dG that accumulated in cells incubated with minimal (25 μg/mL) and maximal (150 μg/mL) concentrations of PtNPs were 100 and ~2000 nm/DNA (μg/μL), respectively, compared with controls. The accumulation of 8-oxo-dG was significantly increased to 100, 200, 400, 800, 1500, and 2000 nm/DNA (μg/μL), respectively, in THP-1 cells incubated with 25, 50, 75, 100, 125, and 150 μg/mL PtNPs. Cells incubated with minimal (25 μg/mL) and maximal (150 μg/mL) concentrations of PtNPs accumulated 200 and ~6000 nm/DNA (μg/μL) of 8-oxo-G, respectively, compared with controls. The accumulation of 8-oxo-G was significantly increased to 200, 400, 800, 1500, 3000, and 6000 nm/DNA (μg/μL), respectively, in THP-1 cells incubated with 25, 50, 75, 100, 125, and 150 μg/mL PtNPs. Notably, significantly more 8-oxo-G than 8-oxo-dG accumulated, indicating that PtNPs target mitochondrial, rather than normal DNA. Zinc oxide NPs induce cellular morphological modifications, mitochondrial dysfunction, reduced SOD, depleted GSH, and oxidative DNA damage in human hepatocyte and embryonic kidney cells [102]. Silicon dioxide NPs (SiO_2_NPs) concentration- and size-dependently increase intracellular ROS, damage DNA, and cause apoptosis in HaCaT cells, and these effects closely correlate with increased oxidative stress [103]. Titanium and silver NPs, as well as ultra-small PtNPs, cause oxidative DNA damage in Cos-1, TK6, human alveolar basal epithelial, and THP-1 cells [22,39,104]. Collectively, these findings correlate with mitochondrial dysfunction. A possible mechanism of 8-oxo-dG and 8-oxo-G accumulation is the overproduction of ROS and RNS that could alter the balance of oxidants and antioxidants that would cause oxidative and nitrative stress and contribute to damaging DNA and RNA.

### 2.11. PtNPs Activate Inflammatory Responses in THP-1 Cells

**Figure 11:**
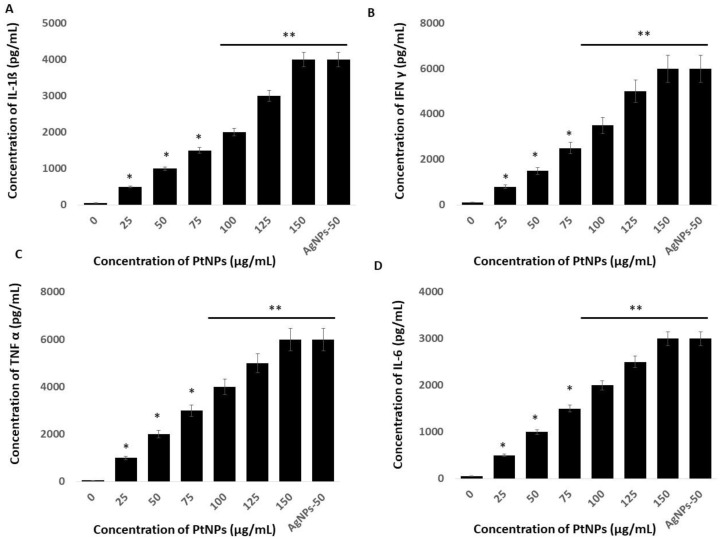
Effect of PtNPs on cytokine and chemokine levels. THP-1 cells were treated with various concentrations of PtNPs (25–150 µg/mL) for 24 h. (**A**) IL-1 beta; (**B**) IFNγ, (**C**) TNFα, and (**D**) IL-6 cytokine concentration was measured in the cell culture supernatant after PtNP treatment. Results are expressed as mean fold change ± standard deviation from three independent experiments. The treated groups showed statistically significant differences from the control group by the Student’s *t*-test (* *p* < 0.05; ** *p* < 0.01). * significant; ** highly significant.

Platinum NPs seem to play critical roles in oxidative stress, mitochondrial dysfunction, and ERS-mediated cell death. We examined whether these events are interconnected, and whether they could induce inflammatory responses in THP-1 cells incubated with PtNPs. The UPR and inflammation might be interconnected through ROS production, calcium release, nuclear factor kappa-light-chain-enhancer of activated B cells (NF-Κb) and mitogen-activated protein kinase (MAPK) activation, and induction of the acute-phase response [105]. To substantiate a relationship between ERS and inflammation, we evaluated levels of the critical cytokines, IL-1β, IFNγ, TNFα, and IL-6 in THP-1 cells incubated with PtNPs for 24 h. Each of these cytokines was significantly induced at all PtNP concentrations. Increasing concentrations of PtNPs significantly increased IL-1β, IFNγ, TNFα, and IL-6 expression (Figure 11). The concentrations of IL-1β were 500 and ~4000 pg/mL pg/mL in cells incubated with minimal (25 μg/mL) and maximal (150 μg/mL) concentrations of PtNPs compared with the control. Fold copy numbers were significantly increased to 500, 1000, 1500, 2000, 3000, and 4000 pg/mL respectively, in THP-1 cells incubated with 25, 50, 75, 100, 125, and 150 μg/mL PtNPs. The concentrations of IFNγ were 800 and ~6000 pg/mL in THP cells incubated with minimal (25 μg/mL) and maximal (150 μg/mL) concentrations of PtNPs compared with the control, and fold copy numbers were increased to 800, 1500, 2500, 3500, 5000, and 6000 pg/mL, respectively, when incubated with 25, 50, 75, 100, 125, and 150 μg/mL PtNPs. The concentrations of TNFα in cells incubated with minimal (25 μg/mL) and maximal (150 μg/mL) concentrations of PtNPs compared with the control were 1000 and ~6000 pg/mL, respectively, and the fold copy number was significantly increased to 1000, 2000, 3000, 4000, 5000, and 6000 pg/mL, respectively, when incubated with 25, 50, 75, 100, 125, and 150 μg/mL PtNPs. The concentrations of IL-6 in cells incubated with minimal (25 μg/mL) and maximal (150 μg/mL) concentrations of PtNPs were 500 and ~3000 pg/mL, respectively, compared with the control, and fold copy numbers were significantly increased to 500, 1000, 1500, 2000, 2500, and 3000 pg/mL, respectively, when incubated with 25, 50, 75, 100, 125, and 150 μg/mL PtNPs. Our findings suggested that oxidative stress caused by ROS and nitric oxide is crucial for integrating inflammatory responses, the ER-stress response, and functional interactions between the ER and mitochondria. Another possible mechanism is that cytokines induced by PtNPs trigger calcium release from the ER and the accumulation of ROS, both of which interfere with protein folding and mitochondrial metabolism [106,107]. 

### 2.12. Gene Expression Profile Is Altered in THP-1 Cells Incubated with PtNPs 

**Figure 12:**
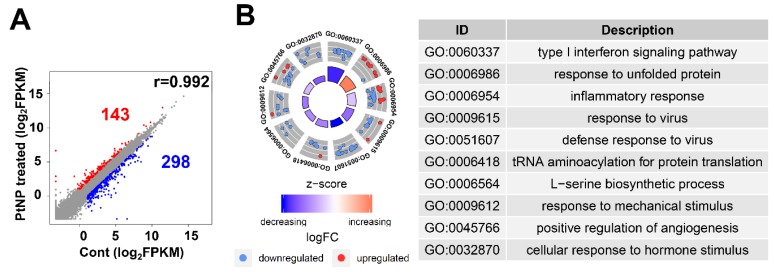
PtNP treatment causes a change in gene expression. (**A**) Scatter plot representing expression profile of PtNP-treated and control samples. The red portion represents upregulated differentially expressed genes (DEGs) and the blue portion represents the downregulated DEGs in the PtNP-treated sample relative to the control sample. (**B**) A plot of gene ontology (GO) circle produced with biological processes of DEGs from the Database for Annotation, Visualization and Integrated Discovery (DAVID). Red dots and blue dots represent upregulated and downregulated genes, respectively. Each GO term is noted with a unique GO number.

Ultra-small PtNPs elicit cytotoxicity by inhibiting cell proliferation, and they decrease cell survival by disrupting mitochondrial functions. Therefore, we used RNA-Seq to analyze the underlying molecular mechanism through which PtNPs reduce cell viability and proliferation in THP-1 cells incubated without (control; *n* = 2) and with PtNPs (*n* = 2). Figure 12A shows that, among 441 differentially expressed genes (DEGs), the expressions of 143 and 298 genes were elevated and repressed, respectively. We investigated the biological consequences of these 441 DEGs using gene ontology (GO) analyses and found that genes associated immune responses (type I interferon signaling pathway and inflammatory responses), various antiviral responses (defense responses to virus and responses to viruses), responses to unfolded proteins, transfer RNA (tRNA) aminoacylation, l-serine biosynthesis, responses to mechanical stimulus, positive regulation of angiogenesis, and cellular response to hormone stimulus were changed (Figure 12B). Given that monocytic THP-1 cells were derived from a patient with acute monocytic leukemia, the differential expression of genes involved in GO biological processes (BP) is reasonable [108]. 

### 2.13. PtNP Treatment Changes Genes Related to Protein Misfolding and Mitochondrial Function

**Figure 13:**
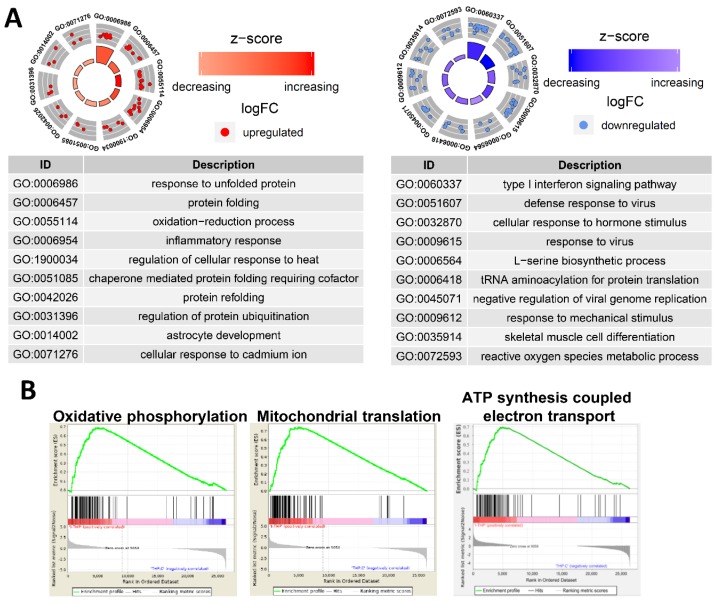
PtNPs alter genes involved in ER and mitochondrial dysfunctions. (**A**). GO circles analyzed with biological processes in DEGs from DAVID. The left circle displays highlighted GO terms of upregulated genes of PtNP-treated sample, and the right circle displays the terms of downregulated genes. Red dots and blue dots represent upregulated and downregulated genes, respectively. Each GO term is noted with a unique GO number. (**B**). Gene set enrichment analyses (GSEA) in the PtNP-treated sample. GSEAs associated with mitochondria dysfunctions are shown.

We analyzed the GO of the up- and downregulated genes. Figure 13A shows that numerous immune-associated genes were repressed in THP-1 cells incubated with PtNPs. Incubation with PtNPs led to the upregulation of proteins involved in protein folding (responses to unfolded protein, protein folding, chaperone-mediated protein folding requiring cofactors, and protein refolding) in THP-1 cells (Figure 13A). Similarly, the downregulated genes were involved in protein synthesis (l-serine biosynthesis and tRNA aminoacylation for protein translation). These data are of interest because charged gold NPs can interact with peptides or proteins to form fibrils [109]. Such an interaction might result in massive protein misfolding that would inhibit cell growth and induce apoptosis. Ultra-small PtNPs also lead to cell apoptosis via mitochondrial dysfunction [18]. Our gene set enrichment analysis (GSEA) revealed that PtNPs induced aberrations in mitochondrial functions including oxidative phosphorylation, mitochondrial translation, and ATP synthesis-coupled electron transport (Figure 13B). 

### 2.14. PtNPs Impair Pathways Involved in Protein Synthesis 

**Figure 14:**
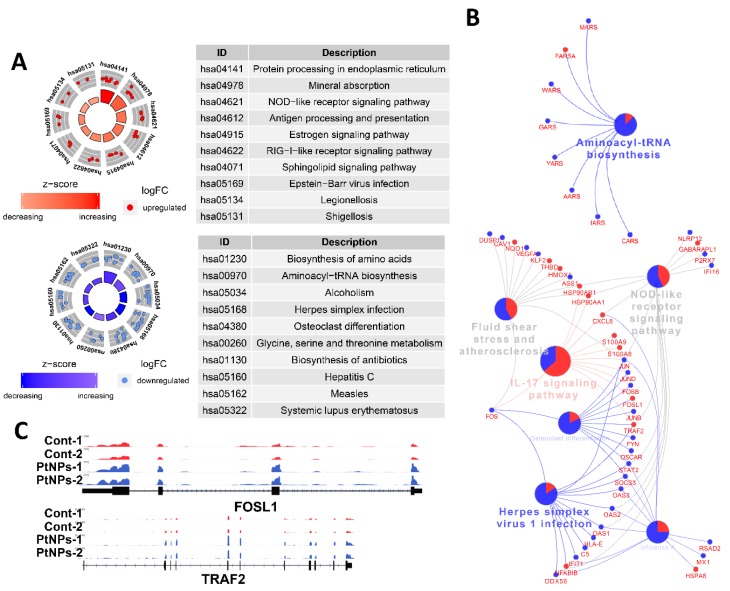
PtNPs impair pathways involved in protein synthesis. (**A**). Kyoto Encyclopedia of Genes and Genomes (KEGG) pathway analysis results of DEGs from DAVID. The upper circle displays highlighted pathways of upregulated genes of PtNP-treated sample, and the lower circle displays the terms of downregulated genes. Note that KEGG pathways related to proteins synthesis are mainly altered. (**B**). KEGG pathway analysis conducted with differentially expressed genes shown in the form of networks. Upregulated genes and downregulated genes are shown in red and blue colors, respectively. Gene networks associated with interleukin-17 (IL-17) signaling pathways and aminoacyl tRNA biosynthesis are changed by PtNPs. (**C**). Representative genes (FOSL1 and EGR1) involved in the IL-17 pathway are visualized.

We applied DEG to Kyoto Encyclopedia of Genes and Genomes (KEGG) pathway analyses to determine which biological pathways were altered by PtNPs. Genes associated with protein synthesis-related KEGG pathways (biosynthesis of amino acids and aminoacyl tRNA) and amino acid (glycine, serine, and threonine) metabolism were changed by PtNPs. When up- or downregulated DEG were applied separately, KEGG pathways of protein processing in the ER and antigen processing and presentation were identified in association with genes that were upregulated by PtNPs, whereas KEGG pathways of protein synthesis were identified in association with those that were downregulated by PtNPs (Figure 14A). Many genes associated with the IL-17 signaling pathway were changed by PtNPs (Figure 14B). Representative genes Fos-like Antigen 1 (FOSL1) and early Growth Response 1 (EGR1) involved in the IL-17 pathway were visualized using the Integrative Genomics Viewer (IGV) genome browser (Figure 14C). The IL-17 pathway plays preventive or promotive roles in some cancers by protecting against viral infection and inducing inflammatory responses [110]. 

### 2.15. Transcriptional Regulation Is Impaired by PtNPs 

**Figure 15:**
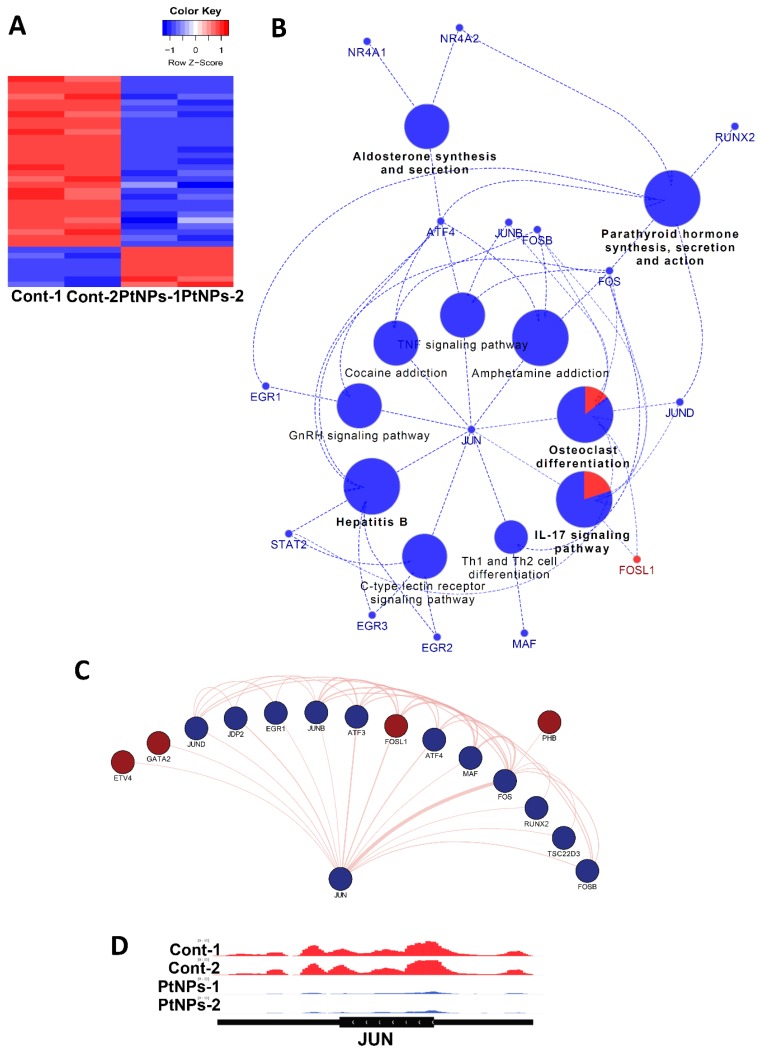
PtNPs change transcriptional networks. (**A**). Heatmap representing relative expression of differentially expressed transcription factors (TFs) in each sample. Seven and 29 TFs are enhanced and repressed by PtNPs. (**B**). KEGG pathway analysis conducted with differentially expressed transcription factors shown in the form of networks. JUN is identified as a key TF-modulating interconnector for other TFs regulating the KEGG pathways. TFs associated with pathways (IL-17 signaling, hormone production and secretion, hepatitis B infection, and T-cell differentiation) are altered. (**C**). Network showing interactions between JUN and other differentially expressed TFs. Dark-red and dark-blue nodes represent transcription factors with relatively higher and lower expression levels, respectively. Edge thickness represents the relative number of research references. (**D**). IGV genome browser images of expression level of JUN gene.

Given the changes in gene expression, we aimed to identify transcriptional factors (TFs) that are aberrantly changed and altered downstream of target genes by PtNPs. Analysis of networks between TFs and their downstream target genes revealed that PtNPs up- and downregulated the expression of seven and 29 TFs, respectively (Figure 15A). When applied to KEGG analysis, biological pathways such as IL-17 signaling, hormones/cytokines synthesis, and secretion and T-cell differentiation were altered (Figure 15B). Finally, interactions among TFs were analyzed, and the results are visualized in Figure 15C. We found that JUN proto-oncogene (JUN) is a key TF-modulating interconnector for other TFs (Figure 15D). Levels of JunB are reduced in association with apoptosis in cultured cells [111]. 

Previously, we demonstrated that apigenin-functionalized ultra-small PtNPs with an average size of 2 nm and significantly spherical in shape caused cytotoxicity in THP-1 cells, whereas lycopene-functionalized PtNPs showed that the anisotropic nature of particles exhibited a variety of shapes such as spherical, cubic, rectangular, triangular, octahedral, and tetrahedral, or truncated cubic, hexagonal, octahedral, and tetrahedral particles with an average size of 25 (range, 5–50) nm. With these particles, the study demonstrated cytotoxicity, apoptosis, inflammatory response, and transcriptomic and molecular pathway alterations in THP-1 cells. This study further described the impact of anisotropic nanoparticles on mitochondrial dysfunction through mitochondrial copy number, PCG-1α expression, and ERS-mediated apoptosis. Interestingly, this study further discussed that lycopene-functionalized PtNPs could change the expression of genes involved in protein misfolding, mitochondrial function, protein synthesis, inflammatory responses, and transcription regulation.

## 3. Materials and Methods 

### 3.1. Materials

The THP-1 cell line obtained from the American Type Culture Collection (ATCC; Manassas, VA, USA). All cells were cultured in 75-cm^2^ tissue culture flasks (Corning Inc., Corning, NY, USA) at 37 °C under a 5% CO_2_ atmosphere and 95% relative humidity. Dried and hydrated hexachloroplatinic acid (H_2_PtCl_6_·6H_2_O) was purchased from Sigma-Aldrich (St. Louis, MO, USA). Penicillin-streptomycin, trypsin- ethylenediaminetetraacetic acid (EDTA), Roswell Park Memorial Institute (RPMI)-1640 cell culture medium, fetal calf serum (FCS), and antibiotic/anti-mycotic reagents were obtained from Life Technologies/Gibco (Grand Island, NY, USA). Toxicology in vitro assay kits were purchased from Sigma-Aldrich. Reagents and kits to measure reactive oxygen species, malondialdehyde (MDA), protein carbonyl content, nitric oxide, and antioxidants, and all other chemicals were purchased from Sigma-Aldrich unless otherwise stated.

### 3.2. Synthesis and Characterization of PtNPs

We synthesized PtNPs by reducing PtCl_6_^2−^ ions to PtNPs in a mixture of 10 mL of 1 mg/mL lycopene and 90 mL of 1 mM aqueous H_2_PtCl_6_∙6H_2_O. Mixtures were maintained for 1 h at 100 °C (using a hotplate) in sealed flasks to avoid evaporation, as increasing temperatures catalyze the reduction process. Identical amounts of platinum solution and lycopene were separately maintained under the same reaction conditions for control experiments. The reduced platinum solution was sonicated for 10 min to separate platinum nanomaterials from biomolecules, then passed through a 0.2-µm syringe filter. The reduced platinum was purified by repeated centrifugation at 5000× *g* for 30 min, and the pellets were washed with distilled water to remove impurities. Purified PtNPs were characterized using UV-visible spectroscopy, X-ray diffraction (XRD), Fourier-transform infrared spectroscopy (FTIR), dynamic light scattering (DLS), and scanning (SEM) and transmission (TEM) electron microcopy.

### 3.3. Cell Culture Conditions and PtNP Exposure

THP-1 cells were cultured in RPMI-1640 cell medium supplemented with 10% FCS, 2 mM l-glutamine, 10 mM HEPES, 1 mM pyruvate, 100 U/mL penicillin, and 0.1 mg/mL streptomycin (Sigma-Aldrich). The cells were sub-cultured usually twice a week with 1 × 10^6^ viable cells/mL and incubated at 37 °C in a 5% CO_2_ atmosphere. The medium was replaced on the following day with 100 µL of fresh medium, and the cells were incubated for 24 h before adding PtNPs. Experiments proceeded in 96-, 24-, and 12-well plates and in 100-mm cell culture dishes, as required. Cells were incubated with various concentrations of PtNPs or required doses of PtNPs.

### 3.4. Cell Viability Assays

Cell viability was measured using cell counting kit-8 (CCK-8; CK04-01l; Dojindo Laboratories, Kumamoto, Japan). Briefly, THP-1 cells were seeded in 96-well flat-bottom culture plates containing various concentrations of PtNPs for 24 h at 37 °C in a humidified 5% CO_2_ incubator. The CCK-8 solution (10 μL) was added to the wells, and the plates were incubated for a further 2 h at 37 °C. Absorbance was measured at 450 nm using a Multiskan FC microplate reader (Thermo Fisher Scientific, Waltham, MA, USA).

### 3.5. BrdU Cell Proliferation Assays

THP-1 cells were incubated with various concentrations of PtNPs for 24 h, and then labeled BrdU was added to the medium 2 h before the end of the incubation. The cells were fixed, and amounts of incorporated BrdU were measured to determine cell proliferation using BrdU enzyme-linked immunosorbent assay (ELISA) kits as described by the manufacturer (Roche Molecular Biochemicals, Mannheim, Germany). The proliferation of THP-1 cells at 0 h was considered as 100%.

### 3.6. Cell Morphology Analysis

THP-1 cells were seeded in six-well plates (2 × 10^5^/well) and incubated with 25–150 μg/mL PtNPs for 24 h. Cells cultured without PtNPs served as the control. Cell morphology was then assessed using an OLYMPUS IX71 optical microscope (Tokyo, Japan) with appropriate filter sets.

### 3.7. Assessment of Membrane Integrity

The membrane integrity of THP-1 cells was evaluated using LDH cytotoxicity detection kits. Briefly, the cells were incubated with various concentrations of PtNPs for 24 h. Subsequently, 100 μL of cell-free supernatant from each well was transferred in triplicate to 96-well plates, and 100 μL of the LDH reaction mixture was added to each well. After a 3-h incubation under standard conditions, the optical density of the final solution was determined at a wavelength of 490 nm using a microplate reader.

### 3.8. Assessment of Dead-Cell Protease Activity 

Dead-cell protease activity was assayed as described [112] in THP-1 cells incubated with various concentrations of PtNPs for 24 h. Protease activity was determined by assessing associations between intracellular proteases and alanyl-alanyl phenylalanine-aminoluciferin, a luminogenic peptide substrate. Luminescence in each well after incubating the substrate (5 μL) for 15 min at 37 °C was measured using a luminescence counter (Perkin Elmer, Waltham, MA, USA). The degree of measured luminescence reflected dead-cell protease activity.

### 3.9. Determination of ROS, MDA, Nitric Oxide (NO), and Carbonylated Protein Levels

We estimated ROS as described previously [18]. THP-1 cells seeded into 24-well plates at a density of 5 × 10^4^/well were cultured for 24 h, then washed twice with phosphate-buffered saline (PBS). Fresh medium containing various concentrations of PtNPs was added, and the cells were incubated for a further 24 h. After supplementation with 20 μM dichloro-dihydro-fluorescein diacetate (DCFH2-DA), the cells were incubated for 30 min at 37 °C and rinsed with PBS. Thereafter, PBS (2 mL) was added to the wells, and fluorescence intensity was determined using a Gemini EM spectrofluorometer (Molecular Devices, Sunnyvale, CA, USA) at excitation and emission wavelengths of 485 and 530 nm, respectively. Levels of MDA determined as thiobarbituric acid-reactive substances were assayed as described with modifications. The production of NO was spectrophotometrically quantified using the Griess reagent (Sigma-Aldrich St. Louis, MO, USA). Absorbance was measured at 540 nm and nitrite concentrations were determined using a calibration curve prepared with sodium nitrite as the standard [18]. Carbonylated protein content was measured as described previously [18].

### 3.10. Measurement of Anti-Oxidative Markers 

The expression of oxidative and anti-oxidative stress markers was determined as described previously [38]. The anti-oxidative stress markers GSH, TRX, CAT, and SOD were quantified according to the manufacturer’s instructions. THP-1 cells were cultured in 75-cm^2^ culture flasks and incubated with various concentrations of PtNPs for 24 h. The cells were scraped into chilled PBS, washed twice with 1 × PBS at 4 °C for six minutes each, and sedimented at 1500 rpm. Cell pellets were sonicated at 15 W for 10 s (three cycles) to obtain cell lysates. Supernatants were stored at −70 °C.

### 3.11. Determination of Mitochondrial Membrane Potential (MMP) 

We measured MMP according to the manufacturer’s instructions (Molecular Probes, Eugene, OR, USA) using the cationic fluorescent indicator, JC-1 (Molecular Probes). THP-1 cells were incubated with various concentrations of PtNPs for 24 h followed by 10 μM JC-1 at 37 °C for 15 min. The cells were washed and resuspended in PBS; then, fluorescence intensity was measured. Mitochondrial membrane potential was expressed as the ratio of the fluorescence intensity of JC-1 aggregates to that of the monomers. 

### 3.12. Measurement of ATP 

THP-1 cells were incubated with various concentrations of PtNPs for 24 h, and then ATP was measured according to the manufacturer’s instructions (Catalog No., MAK135; Sigma-Aldrich St. Louis, MO, USA). Decreased levels of ATP indicated increased cytotoxicity. 

### 3.13. Analysis of Mitochondrial DNA Copy Number 

Mitochondrial dysfunction analysis was analyzed using real-time PCR amplification and by assessing mitochondrial copy numbers in THP-1 cells incubated with various concentrations of PtNPs for 24 h. The following primers were used to determine copy numbers: mtDNA forward, CCTATCACCCTTGCCATCAT; mtDNA reverse, AGGCTGTTGCTTGTGTGAC. Nuclear DNA was quantified using the following primer set that detects the Pecam gene on chromosome 6: forward, ATGGAAAGCCTGCCATCATG; reverse, TCCTTGTTGTTCAGCATCAC [3].

### 3.14. Reverse Transcription-Quantitative Polymerase Chain Reaction (RT-qPCR)

THP-1 cells were incubated with various concentrations of PtNPs for 24 h; then, total RNA was extracted using PicoPure RNA isolation kits (Arcturus Bioscience, Mountain View, CA, USA). Samples were prepared according to the manufacturer’s instructions. RT-qPCR proceeded using a Vill7 device (Applied Biosystems, Foster City, CA, USA) and the double-stranded (ds)DNA-specific fluorescent dye SYBR Green (Applied Biosystems). Target gene expression was normalized to that of glyceraldehyde-3-phosphate dehydrogenase (*GAPDH)*, which was not affected by PtNPs. 

### 3.15. Measurement of 8-Oxo-7,8-dihydro-20-deoxyguanosine (8-Oxo-Dg) and 8-Oxo-G Levels

THP-1 cells were incubated with various concentrations of PtNPs for 24 h; then, 8-oxo-dG content was determined as described previously [18] and as per the manufacturer’s instructions (Trevigen, Gaithersburg, MD, USA). We prepared 8-OHdG and 8-OHG precoated well strips and then equilibrated them with all reagents at room temperature. Thereafter, either horseradish peroxidase (HRP)-conjugated 8-OHdG or HRP-conjugated 8-OHG antibody was added to standards or samples. All unbound reagents were removed after a 60-min incubation; then, absorbance was measured at 450 nm using an ELISA plate reader.

### 3.16. Measurement of Cytokines 

THP-1 cells were incubated with PtNPs for 24 h, and cytokines were evaluated using a multianalyte inflammatory cytokine ELISA array (Qiagen, Hilden, Germany). Sample values were normalized to control values and displayed as fold changes over control.

### 3.17. Library Preparation and Sequencing

THP-1 cells were incubated with IC_50_ concentrations of PtNPs for 24 h. Total RNA was isolated from control or treated cells using TRI reagent (Merck, Darmstadt, Germany). In total, 500 ng of total RNA was processed for the preparation of the whole-transcriptome sequencing library. Depletion of ribosomal RNA (rRNA) was performed using an MGIEasy RNA Directional Library Prep Kit (MGI Tech, Shenzhen, China) according to the manufacturer’s instruction. The total RNAs were fragmented and copied into first-strand complementary DNA (cDNA) using reverse transcriptase and random primers. Strand specificity was achieved in the RT directional buffer, followed by second-strand cDNA synthesis. Subsequently, the cDNA fragments were ligated to sequencing adapter. The products were then purified and enriched with PCR amplification. The double-stranded library was quantified using a QauntiFluor ONE dsDNA System (Promega, Madison, WI, USA). The library was cyclized at 37 °C for 60 min, and digested at 37 °C for 30 min, followed by cleanup of the circularization product. To make a DNA nanoball (DNB), the library was incubated at 30 °C for 25 min using a DNB enzyme. Finally, the library was quantified by a QauntiFluor single-stranded (ss)DNA System (Promega, Madison, WI, USA) and sequenced on the MGIseq system (MGI Tech, Shenzhen, China) with 100-bp paired-end reads.

### 3.18. Bioinformatics

Using the STAR (v2.6.1b, Cold Spring Harbor, NY, USA) tool, the sequencing reads were aligned and mapped to the UCSC human hg38 genome assembly. DEGs were obtained using Cuffnorm of Cufflinks (v2.2.1, Seattle, WA, USA). Scatter plot and gene ontology (GO) obtained using either DAVID (v6.8) or GOplot (v1.0.2, MLL Münchner Leukämielabor GmbH, Munich, Germany) tools were plotted in R package (v3.6.1). GSEA (v4.0.1, BROAD Institute, Cambridge, MA, USA) analysis was performed to show any phenotypic differences between control and PtNP treatment. IGV (v2.5.3, BROAD Institute, Cambridge, MA, USA) was used to visualize expression levels of DEGs. Transcription factors were determined with data from HumanTFDB (v3.0, Wuhan, China). Networks of transcription factors were analyzed in GeneMANIA tool (University of Toronto, ON, Canada). The heatmap was produced with the heatmap2 function of gplots (v3.0.1.1, Boehringer Ingelheim, Ingelheim, Germany) in R package. KEGG pathway analysis was performed using the ClueGO (v2.5.1, INSERM, Paris, France) plug-in for Cytoscape (v3.6.1, Institute of Systems Biology, Seattle, WA, USA). Protein-protein interactions were shown using the iRegulon (v1.3, Leuven, Belgium) plug-in for Cytoscape (v3.6.1, Institute of Systems Biology, Seattle, WA, USA).

### 3.19. Statistical Analysis

Independent experiments were repeated at least three times such that data are represented as means ± standard deviation (SD) for all replicates within an individual experiment. Data were analyzed using the *t*-test, multivariate analysis, or one-way analysis of variance (ANOVA), followed by Tukey’s test for multiple comparisons to determine the differences between groups denoted by an asterisk. The GraphPad Prism analysis software was used for all analyses.

## 4. Conclusions

Due to the immense growth of nanotechnology, PtNPs are widely applied due to their unique plasmonic properties. However, hazardous features and cytotoxicity to human cells, particularly monocytic cell lines, limited the medical and biological applications of PtNPs. Hence, we prepared anisotropic rather than homogeneous PtNPs using lycopene. We then investigated the effects of these PtNPs on THP-1 cells to determine their potential applications to consumer products. Our results showed that PtNPs potentially cause oxidative stress, which leads to a loss of cell viability, decreased proliferation, the induction of ROS, the upregulation of MDA, NO, protein carbonylation, ERS, apoptosis, and proinflammatory responses. Platinum NPs induced damage to the mitochondrial membrane potential, ATP synthesis, mitochondrial copy numbers, and biogenesis due to the generation of excess intracellular ROS. Excessive oxidative stress eventually caused an imbalance between intracellular pro- and antioxidants. Notably, PtNPs induced ERS-mediated cell death by increasing the expression of IRE1, PERK, ATF6, and ATF4, the apoptotic markers, p53, Bax, caspase 3, and the anti-apoptotic marker Bcl-2. These results indicated that PtNPs activate ERS, mitochondrial-mediated apoptosis, and proinflammatory responses by inducing the expression of IL-1β, IFNγ, TNFα, and IL-6. Transcriptomic and molecular pathway analyses of THP-1 cells incubated with at IC_50_ concentrations of PtNPs showed the expression of genes involved in protein misfolding, mitochondrial function, protein synthesis, inflammatory response, and transcription regulation. All these data were significantly associated with the findings of previous studies in vitro. Overall, our findings comprise a resource of transcriptional information upon which to base further mechanistic studies of PtNP toxicity. Collectively, PtNPs induce cell death through a specific mechanism of toxicity involving oxidative stress, mitochondrial dysfunction, ERS, and proinflammatory responses. Importantly, our genome-scale analysis reflected the phenotypes of THP-1 cells incubated with PtNPs in vitro. The proposed hypothesis confirmed the cytotoxic, apoptosis, and proinflammatory effects of anisotropic nanoparticles comparable with spherical nanoparticles. Mostly, anisotropic nanoparticles showed the capacity for tenacious and longer-term blood circulation, as well as higher in vivo targeting specificity, than their spherical counterparts. These features of anisotropic nanoparticles could enable the technology as an attractive platform for nanomedicine in biomedical applications. The toxic models of nanoparticles are tightly linked to the development of nanomedicines. Whereas uncontrolled exposure of human systems leads to toxicity, the selective induction of cytotoxicity in cancer cells could help to develop safe therapy for cancer. The increasing understanding of nanotoxicity studies would help to develop the safe design of nanomaterial-based cancer therapy. In particular, lycopene-functionalized PtNPs could change the expression of genes involved in protein misfolding, mitochondrial function, protein synthesis, inflammatory responses, and transcription regulation. These features of anisotropic nanoparticles could enable the technology as an attractive platform for nanomedicine in biomedical applications.
